# Infection of *Beet necrotic yellow vein virus* with RNA4-encoded P31 specifically up-regulates pathogenesis-related protein 10 in *Nicotiana benthamiana*

**DOI:** 10.1186/1743-422X-11-118

**Published:** 2014-06-24

**Authors:** Wen-Qi Wu, Hui-Yan Fan, Ning Jiang, Ying Wang, Zong-Ying Zhang, Yong-Liang Zhang, Xian-Bing Wang, Da-Wei Li, Jia-Lin Yu, Cheng-Gui Han

**Affiliations:** 1 State Key Laboratory of Agrobiotechnology and the Ministry of Agriculture Key Laboratory for Plant Pathology, China Agricultural University, Beijing 100193, China; 2 Department of Plant Pathology, China Agricultural University, Beijing 100193, China

**Keywords:** *Beet necrotic yellow vein virus*, RNA4, P31, Pathogenesis-related protein 10, *Nicotiana benthamiana*

## Abstract

**Background:**

*Beet necrotic yellow vein virus* (BNYVV) is the infectious agent of sugar beet rhizomania, which consists of four or five plus-sense RNAs. RNA4 of BNYVV is not essential for virus propagation in *Nicotiana benthamiana* but has a major effect on symptom expression. Early reports showed that RNA4-encoded P31 was associated with severe symptoms, such as curling and dwarfing, in *N. benthamiana*.

**Results:**

We discovered that the pathogenesis-related protein 10 (PR-10) gene can be up-regulated in BNYVV-infected *N. benthamiana* in the presence of RNA4 and that it had a close link with symptom development. Our frame-shift, deletion and substitution analysis showed that only the entire P31 could induce *PR-10* up-regulation during BNYVV infection and that all the tryptophans and six cysteines (C174, C183, C186, C190, C197 and C199) in the cysteine-rich P31 had significant effects on *PR-10* expression. However, P31 could not interact directly with PR-10 in yeast.

**Conclusions:**

Our data demonstrated that only integrated P31 specifically induced *PR-10* transcription, which coincided closely with the appearance of severe symptoms in BNYVV-infected *N. benthamiana*, although they could not interact directly with each other in yeast.

## Introduction

*Beet necrotic yellow vein virus* (BNYVV) [1] is a member of the *Benyvirus* genus, which causes rhizomania disease of sugar beet (*Beta vulgaris* L.) transmitted by the soil-inhabiting plasmodiophorid, *Polymyxa betae*[2]. Rhizomania [3] is one of the most economically important diseases affecting sugar beet, and it is widespread in all of the sugar beet-growing areas of Europe, Asia and America [4]. The disease is characterized by abnormal rootlet proliferation and reduced sugar yields [5]. The BNYVV genome consists of four or five plus-sense 5′-capped and 3′-polyadenylated RNAs [6,7]. RNA1 and RNA2 carry ‘house-keeping’ genes that are involved in virus replication, assembly, cell-to-cell movement and the suppression of post-transcriptional gene silencing [8-11]. Extra genomic components, consisting of smaller RNA3, RNA4 and RNA5, each of which plays an important but unique role in pathogenicity and vector transmission are necessary for the efficient production of typical rhizomania symptoms, long-distance movement and vector propagation [6,10,12-15]. The RNA3-encoded P25 protein is responsible for the virulence and avirulence factors in leaves of some resistant sugar beet lines [16]. The P25 protein also functions in the induction of rhizomania symptoms of sugar beet roots and the severe symptom expression in the Chenopodiaceae hosts [17-21]. The RNA5-encoded 26-kDa protein (P26), which is associated with symptom severity but is dispensable for BNYVV survival, has been found in small areas of Europe and in most areas of Asia [4,22,23].

In the case of BNYVV, the presence of the RNA4-encoded single 31-kDa protein (P31) is involved in enhanced symptom expression, efficient root-specific silencing suppression and efficient vector transmission by *P. betae*[13]. BNYVV induces plant stunting, downward curling of the upper leaves and severe mosaicism with leaf distortions in the systemic host *Nicotiana benthamiana*[24] without affecting viral RNA accumulation [2,13]. The entire P31 is required for the expression of severe symptoms in *N. benthamiana*[13].

When a pathogen attacks a plant, it displays an innate pathogen-specific resistance by producing responses, including oxidative bursts of cells, changes in cell wall composition and the synthesis of compounds like phytoalexin and pathogenesis-related proteins (PRs). PRs are a major category of host proteins induced during biotic and abiotic stresses, which have been identified in 17 different families thus far in monocotyledonous and dicotyledonous plants based on their structural and functional properties [25-28]. The PR-10 family plays an important role among the PR groups. RNase activity has been reported for some PR-10 proteins and may be involved in its antimicrobial activity [29].

Thus, we investigated gene transcription levels in *N. benthamiana* infected by different BNYVV isolates, and we found that RNA4 could dramatically up-regulate *PR-10* expression and was closely linked to symptom appearance. Frame-shift and deletion mutations in the P31-encoding region indicated that only an entire intact P31 could induce PR-10 up-regulation. An amino acid substitution analysis of the P31 protein showed that several cysteine/tryptophan positions affected *PR-10* expression and symptom development during BNYVV infection.

## Results

### BNYVV coupled with RNA4 specifically induces *PR-10* and *PR-Q* up-regulation in *N. benthamiana*

**Figure 1:**
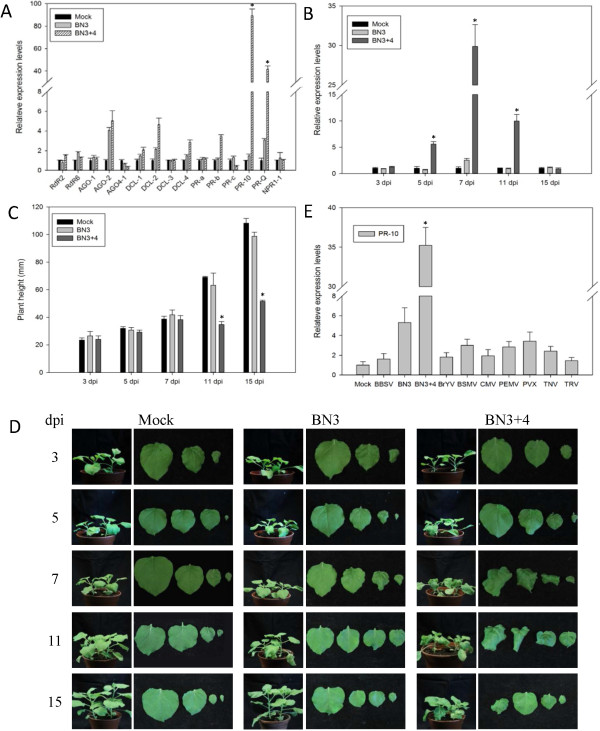
**Gene expression levels of *Nicotiana benthamiana *infected by *Beet necrotic yellow vein virus *(BNYVV) RNAs 1, 2, 3 and 4 (BN3 + 4) and a time-course investigation of*PR-10*and symptoms.** Normalization of expression was performed using the *PP2A* gene as the internal reference. Data are presented as means ± Standard Error of the Mean (SEM), *P < 0.05; n = 4/group. A control mock-inoculated sample was used as the calibrator (=1). Each experiment was repeated at least two times. **(A)** Expression levels of genes related to gene silencing and pathogenesis in plants at 11 dpi. **(B)** Time-course of *PR-10* expression levels in *N. benthamiana* infected by BNYVV RNAs 1, 2 and 3 (BN3) and BN3 + 4 at 3, 5, 7, 11 and 15 dpi. **(C)** Mean height of tested plants at different times. **(D)** Typical symptoms on *N. benthamiana*. The top three or four leaves were collected. **(E)***PR-10* expression levels in *N. benthamiana* plants inoculated with several plant viruses.

Previous studies showed that *N. benthamiana* inoculated with a BNYVV isolate without RNA4 (BN3, which contains BNYVV RNAs 1, 2 and 3) does not show severe symptoms, whereas a BNYVV isolate with RNA4 (BN3 + 4, which contains BNYVV RNAs 1, 2, 3 and 4) induces a downward curling of the upper leaves at 10–12 days post inoculation (dpi) in *N. benthamiana*. The plant is stunted and these first leaves that undergo curling gradually wilt [13,30]. After 15 dpi, the newly-grown leaves recovered in plants infected by BN3 without RNA4 [30]. Plant development is regulated by many genes in different situations, such as biotic stresses. The relative expression levels of select genes, involved with gene silencing and pathogenesis, were determined using quantitative real-time PCR (qPCR) at 11 dpi. The primers used in detection are listed in Table 1. The genes related to gene silencing were expressed at similar levels in plants infected with BN3 and BN3 + 4 (Figure 1A), except for DCL-2, which was expressed at twice the level with BN3 + 4 than with BN3. *PR-a*, *PR-c* and *NPR1-1* expression levels were not different between the test plant types, although *PR-b* was up-regulated less than three-fold with BN3. *PR-Q* and *PR-10* were up-regulated by tens of folds, and *PR-10* had at least a 30-fold increased expression level in BN3 + 4 compared with BN3. Thus, BN3 + 4 specifically induced *PR-10* and *PR-Q* up-regulation in *N. benthamiana*, and *PR-10* had the highest expression level.

**Table 1:** Sequences of primers for qRT-PCR

**Primer**	**Sequence (5′ − 3′)**
RDR2_FOR	CATAAGAAATTGGCATCGGCG
RDR2_REV	TCCAGCAAAATGTCACCTACC
RDR6_FOR	CTTTGGATGAGAAGTGCCTA
RDR6_REV	TTTGGGACAAGCTCAAGTC
AGO1_FOR	GCTCTAGAAGATCTGTACAAGACTTGGC
AGO1_REV	CGAATTCTTATTGGCAAACAACCTAGT
AGO2_FOR	TAACATGGTGTTTGTGCGTGAATC
AGO2_REV	CAGTGGCTCTCAAAAGACCAAAGT
AGO4-1_FOR	TATATCCATTGTGGCTCCGGTAAG
AGO4-1_REV	AGAAACATTTTCCTGAAGTCGAGGC
DCL1_FOR	TGTGGGTGATGCAGTATT
DCL1_REV	TGAAACCTGGTTTTGATAGT
DCL2_FOR	CGGGATCC_CCGGGATTTATTCGTAAT
DCL2_REV	CCCTCGAG_AATGACAAAGCCGCTACT
DCL3_FOR	ACTTGTTGAATGCGGTGAAG
DCL3_REV	CCCCTGTCGTTCTAGCTCAT
DCL4_FOR	CGTCCGTGCCCAGAAATCT
DCL4_REV	AATGCAATTGCCGCTTTGA
PRa_FOR	TGTGTTCCTGTTGCTGAATGTT
PRa_REV	GGCGTTGGAATAATGAAGGTAG
PRb_FOR	GGCCTCCAAGCAATTCTCCTCT
PRb_REV	CCATTTAAAGGACATGCTGCTAC
PRc_FOR	GGGCTGATGAACAAAAGTATTA
PRc_REV	AACATCACCACTCTCACAAACA
PR-10_FOR	GAAGAAGAACACAATGAAGGCA
PR-10_REV	CAGTAGGATTGGCAAGAAGGTA
PR-Q_FOR	TTGTTCGGCAAAATGACCAG
PR-Q_REV	TGTGGCTACTAAATCAGGGTT
NPR1-1_FOR	AAAACCTCAGCAAGGAACGCTA
NPR1-1_REV	TGTTGGCCTATTTGTATAGTGGA
PP2A_FOR	GACCCTGATGTTGATGTTCGCT
PP2A_REV	GAGGGATTTGAAGAGAGATTTC

To examine whether other plant viruses could induce *PR-10* up-regulation, *N. benthamiana* was inoculated with several plant viruses, including *Beet black scorch virus* (BBSV), Brassica yellows virus (BrYV), *Barley stripe mosaic virus* (BSMV), *Cucumber mosaic virus* (CMV), *Pea enation mosaic virus* (PEMV), *potato virus X* (PVX), *Tobacco necrosis virus* (TNV) and *Tobacco rattle virus* (TRV). We detected PR-10 expression levels in all the tested plants when symptoms of the corresponding viruses appeared on leaves at 7–14 dpi. However, the PR-10 expression levels were not up-regulated when *N. benthaminana* plants were inoculated with these viruses, and the *PR-10* expression level increased, at most, by three-fold. This is far less than was seen with the BN3 + 4 inoculations, indicating that the high level of *PR10* up-regulation in *N. benthamiana* was BNYVV isolate specific (Figure 1E). Of course, it is possible that there are other plant viruses that could up-regulate PR-10 gene expression levels.

### Time-course analysis showed that increased expression of the PR-10 gene is closely linked to the appearance of severe symptoms in the presence of RNA4

To confirm *PR-10* expression characteristics during a BN3 + 4 infection, we examined the expression levels of the PR-10 gene, plant height and leaf curling at 3, 5, 7, 11 and 15 dpi. Initially, all plants were investigated using a western blot analysis with antiserum specific to the viral coat protein to confirm that plants were systemically infected by BNYVV (data not shown). The *PR-10* expression level was determined by qPCR, and the expression curve was parabola shaped. At 5 dpi, the *PR-10* expression level in plants infected with BN3 + 4 was slightly higher than with BN3, reaching the peak of the parabola at 7 dpi. Then, expression decreased, but was still higher than with BN3, and the level of *PR-10* returned to normal by 15 dpi (Figure 1B). The leaves of plants inoculated with BN3 + 4 were slightly distorted at 5 dpi, compared with those infected with BN3, and the newly-growing leaves showed severe symptoms at 7 dpi. The plant heights showed the largest disparities by 11 dpi when the stunting of plants infected with BN3 + 4 became apparent. The neonatal leaves recovered and appeared flat at 14 dpi. After 15 dpi, newly-growing stems in plants inoculated with BN3 appeared normal (Figure 1C and D).

### First initiation codon of the BNYVV P31 gene is required to induce *PR-10* expression

**Figure 2:**
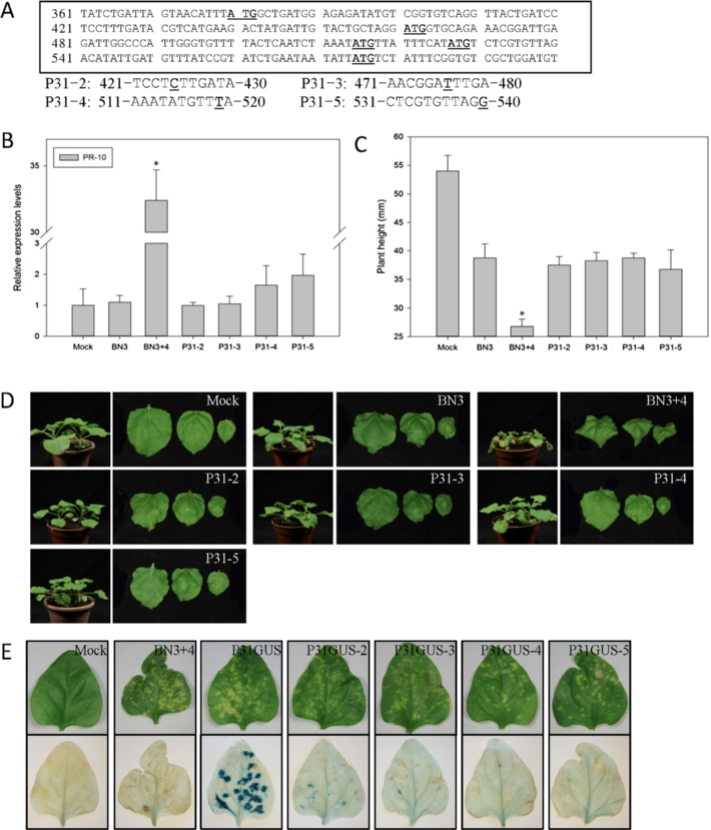
***PR-10 *expression and symptoms of *Nicotiana benthamiana *plants infected by RNA4 different AUG frame-shift mutants plus *Beet necrotic yellow vein virus *(BNYVV) RNAs 1, 2 and 3 (BN3).** This experiment was repeated three times. Data are presented as means ± SEM. *P < 0.05; n = 4/group. **(A)** The 5′−leader nucleotide sequence of the P31 gene and the different frame-shift mutants. **(B)***PR-10* expression levels in *N. benthamiana* plants. A control mock-inoculated sample was used as the calibrator (=1). **(C)** Mean height of tested plants. **(D)** Typical symptoms on leaves of tested plants. **(E)** Phenotype of *Tetragonia expansa* infected by the mutants plus BN3 and GUS histochemical staining.

The open reading frame (ORF) of P31 contains five potential initiation codons. To identify the translational start codon, the P31 gene nucleotide sequence was modified to produce a series of frame-shift mutants (Figure 2A). Four frame-shift mutations (P31-2, −3, −4 and −5) were created after each initiation codon, which abolished the synthesis of P31 proteins initiated at the front AUGs. *N. benthamiana* was inoculated with BN3 alone, RNA4 wild type (WT) or each mutants plus BN3. The expression level of *PR-10* was examined and no significant differences were observed among BN3 and the mutants (Figure 2B), suggesting that *PR-10* up-regulated expression required the entire P31 protein. All plants inoculated with the mutants showed mild symptoms similar to those for BN3, contrasting with the severe symptoms induced by BN3 + 4 at 11 dpi (Figure 2C and D).

To confirm P31 expression, a GUS gene without a start codon was fused to the P31 gene in RNA4 to produce P31GUS. Frame-shift mutants were also constructed and named P31GUS-2, −3, −4 and −5. *Tetragonia expansa* leaves were inoculated with a mixture of WT and each mutant transcript together with BN3, and every inoculated leaf produced similar local lesions at 7 dpi [31]. Histochemical GUS staining of the leaves collected from the different treatments revealed the expression of the fused proteins. Blue spots were observed on leaves inoculated with P31GUS, P31GUS-2 and −3, but the latter two frame-shift mutants produced less and smaller spots than the WT (Figure 2E). This comparative analysis permitted us to conclude that P31 translation initiation mainly occurred at the first AUG, but the second and third AUG may weakly express an incomplete protein that could not induce *PR-10* expression nor severe symptoms in *N. benthamiana*.

### Spontaneous deletions in the P31 gene coding region result in the loss of induced *PR-10* expression

**Figure 3:**
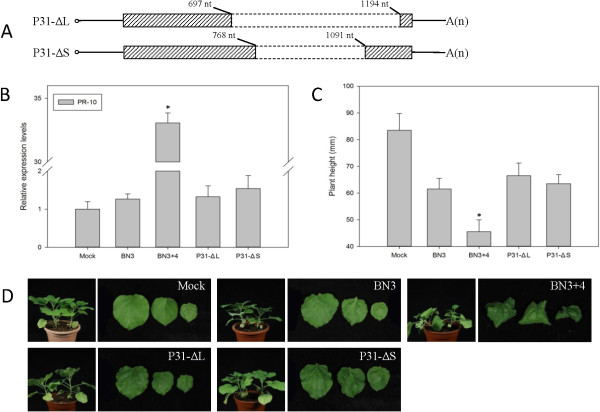
***PR-10*expression and symptoms of *Nicotiana benthamiana *plants infected by RNA4 spontaneous deletion mutants plus *Beet necrotic yellow vein virus *(BNYVV) RNAs 1, 2 and 3 (BN3).** Data are presented as means ± SEM. *P < 0.05; n = 4/group. This experiment was repeated two times. **(A)** Constructs of spontaneous deletion mutants according to published reports. **(B)***PR-10* expression levels of tested plants. A control mock-inoculated sample was used as the calibrator (=1). **(C)** Mean height of tested plants. **(D)** Typical symptoms on leaves of tested plants.

BNYVV isolates maintained by mechanical inoculation into leaves of hosts often accumulate specific forms of RNA-4 deletion mutants [32,33]. On the basis of reported spontaneous deletion sequences, two deletion mutants were created: P31-ΔL and -ΔS. When comparing the spontaneous deletion mutants with WT, P31-ΔL showed the largest deletion region, aa 107–272, and P31-ΔS the smallest, aa 130–238, as was reported for mutants isolated from France [32-34]. The deletion regions, nt 697–1194 and nt 768–1091, were located in the 3′-half of the P31 gene (Figure 3A). The *PR-10* expression levels for BN3, P31-ΔL and -ΔS did not differ compared with WT (Figure 3B). Plants infected with the mutants had mild symptoms, such as leaf slight distortions, but little was distinguishable from the symptoms induced by BN3 (Figure 3C and D). Although the spontaneous deletion mutants did not affect the replication of BNYVV [34], they affected the expression of *PR-10* and attenuated the pathogenicity of P31.

### P31 is a cysteine-rich protein and most cysteines (Cys) are required for *PR-10* up-regulation

**Figure 4:**
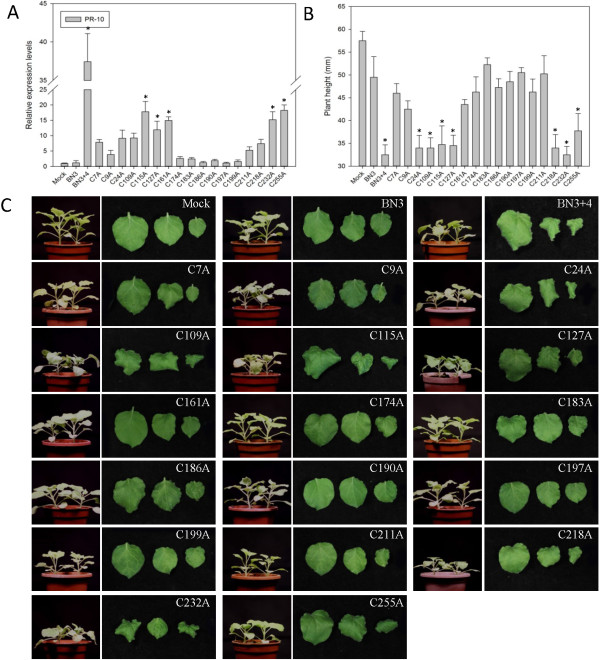
***PR-10 *expression and symptoms of *Nicotiana benthamiana *plants infected with RNA4 cysteine substitution mutants plus *Beet necrotic yellow vein virus *(BNYVV) RNAs 1, 2 and 3 (BN3).** Data are presented as means ± SEM. *P < 0.05; n = 4/group. This experiment was repeated three times. **(A)***PR-10* expression levels of plants. A control mock-inoculated sample was used as the calibrator (=1). **(B)** Mean height of tested plants. **(C)** Typical symptoms on leaves of test plants.

Interestingly, the sequence analysis showed that P31 was rich in Cys and compared with the spontaneous deletion mutants mentioned above, most of the **Cys** were located in the deletion region. To examine the impact of these cysteines on *PR-10* induction, alanine (Ala) scanning (substitution of Ala for Cys) was conducted for all of the Cys (C7A, C9A, C24A, C109A, C115A, C127A, C161A, C174A, C183A, C186A, C190A, C197A, C199A, C211A, C218A, C232A and C255A). Transcripts of each mutant mixed with BN3 were separately inoculated into five unfolded leaves of *N. benthamiana*, and different phenotypes were observed after 11 dpi. *PR-10* expression levels were analyzed, and most expression patterns were in accordance with the degree of symptoms. Compared to *PR-10* expression levels in plants inoculated with BN3, six Cys mutants (C174A, C183A, C186A, C190A, C197A and C199A) induced the same expression level, six Cys mutants (C7A, C9A, C24A, C109A, C211A and C218A) induced a 4- to 7-fold increase and five (C115A, C127A, C161A, C232A and C255A) induced a 10- to 15-fold increase (Figure 4A). At the same time, the severe symptoms in six substitution mutants positioned in P31^174–199^ disappeared compared with the WT. Four mutants (C7A, C9A, C161A and C211A) could still induce the down curling and distortion of upper leaves, but to a lesser degree than the WT. The remaining mutants (C24A, C109A, C115A, C127A, C218A, C232A and C255A) had the same leaf shape as WT. Only C24A, C109A, C115A, C127A, C218A, C232A and C255A mutants resulted in stunted plants, but others were similar to plants infected with BN3 (Figure 4B and C). These results indicated that some Cys in P31 were required for inducing *PR-10* expression, and most of them (C174, C183, C186, C190, C197 and C199) were located in the cysteine-rich region. However, the effects of Cys on *PR-10* and disease symptom expression differed.

### Tryptophans (Trp) in P31 are also important for inducing *PR-10* up-regulation

**Figure 5:**
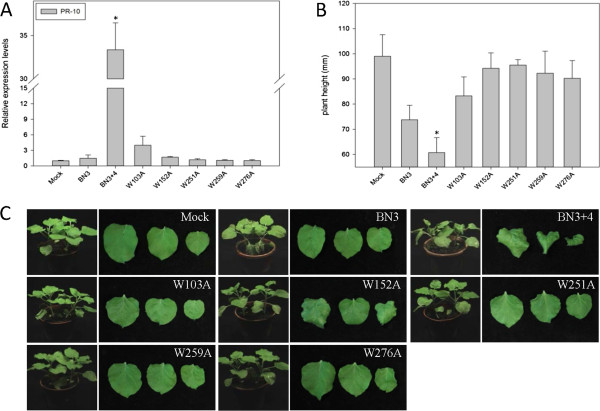
***PR-10 *expression and phenotypes of *Nicotiana benthamiana *plants infected by RNA4 tryptophan substitution mutants plus *Beet necrotic yellow vein virus *(BNYVV) RNAs 1, 2 and 3 (BN3).** Data are presented as means ± SEM. *P < 0.05; n = 4/group. This experiment was repeated three times. **(A) ***PR-10* expression levels of the plants. A control mock-inoculated sample was used as the calibrator (=1). **(B)** Mean height of tested plants. **(C)** Typical symptoms on leaves of tested plants.

P31 has a root-specific silencing suppression function, and recent studies showed that some viral suppressors of RNA silencing mimic the host glycine/tryptophan protein to prevent gene silencing [35-38]. P31 contains five Trp, and there is a glycine before the first and third Trp, respectively. Thus, we used Ala to replace Trp 103, 152, 251, 259 and 276. After 12 dpi, *PR-10* expression levels in the test plants infected with the mutants appeared for the same as those infected with BN3 (Figure 5A). Symptoms were not observed except for with the W152A mutant, which showed a slight distortion of the upper leaves (Figure 5B and C). Our mutation analyses revealed that every Trp in P31 was important for inducing *PR-10* up-regulation and symptom development.

### P31 cannot interact directly with *PR-10* in a yeast system

**Figure 6:**
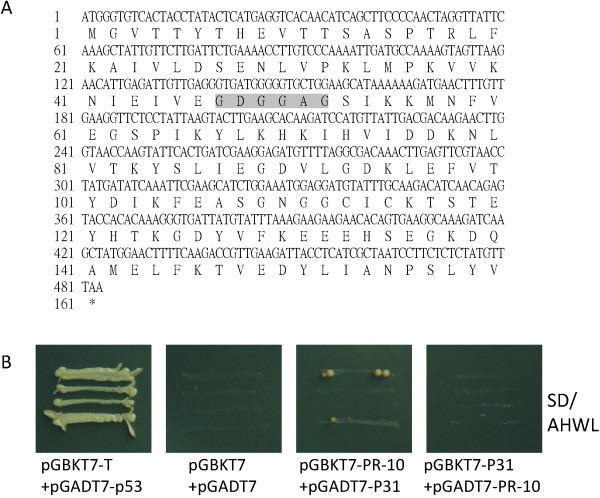
**Nucleotide and protein sequences of NbPR-10 and interactions of P31 and NbPR-10 in the Yeast Two-hybrid (YTH) system. (A)** Nucleotide and deduced amino acid sequence of NbPR-10 cDNA (GenBank ID: KF841443). The P-loop motif is shaded. **(B)** YTH assay of P31 and NbPR-10 interactions, indicating that they have no direct interaction. This experiment was repeated two times.

The PR-10 gene of *N. benthamiana* (*NbPR10*) was cloned by RT-PCR, and it was 483 bp long and capable of encoding a 160-aa peptide. There was a typical GxGGxG motif at 47–52 aa, which is known as the phosphate-binding loop (P-loop), which is frequently found in protein kinases as well as in nucleotide-binding proteins (Figure 6A) [30]. A yeast two-hybrid (YTH) assay was performed to determine whether NbPR-10 and P31 had any intrinsic ability to interact. The preliminary results indicated that they could not interact directly in yeast (Figure 6B).

## Discussion

In the presence of RNA-1 and −2, RNA4 induces leaf stunting, curling and severe mosaicism with leaf distortions in *N. benthamiana* without affecting viral RNA accumulation [39]. BNYVV p31 plays a multifunctional role in efficient vector transmission, enhanced symptom expression and root-specific silencing suppression [13]. Here, we demonstrated that RNA4 drastically up-regulated the expression of PR-10 and PR-Q genes, but the silencing-associated genes had the same expression levels as in *N. benthamiana* infected with BN3. A time-course detection showed that the up-regulation of *PR-10* gene in the presence of RNA4 was coincided closely with the appearance of symptoms, and that the *PR-10* expression occurred slightly before the symptoms.

The PR-10 family is one of the most important families among the PR groups. Unlike most PRs, PR-10 proteins are typically intracellular, small and acidic, with similar three-dimensional structures [26,40-42]. *In vitro PR-10* proteins have been reported to have ribonuclease activity, enzymatic activities in plant secondary metabolisms and roles in abiotic stresses [26]. The *PR-10* family is structurally related to ribonucleases [29,43,44]. As reported, there is a strikingly conserved sequence motif GxGGxG at residues aa 47–52 throughout the family of intracellular PR proteins [45]. This same motif also occurred in the *NbPR-10* from *N. benthamiana*. This motif is frequently found in protein kinases and nucleotide-binding proteins [46].

A few viruses can trigger a *PR-10* response in their host [43,47-49], but the capability to cleave viral RNA remains to be determined. Some PR-10s have their strongest expression in root tissue [40,50,51]. BNYVV causes rhizomania disease of sugar beet, which is characterized by excessive growth of lateral roots and constriction of the taproot, and RNA4-encoded P31 is involved in root-specific silencing suppression. Our results showed that, in BN3 + 4 infected plants, *PR-10* expression and symptom development occurred at similar times. This is somewhat surprising given that *PR-10* is associated with inducing symptoms. However, the experiment showed that P31 could not interact directly with PR-10. Thus, P31, like P25, which interacts with transcription factors [52], works with some factors in the process of *PR-10* expression. Of course, we cannot rule out the possibility that the increased PR-10 level is a response to BN3 + 4 infection, although it failed to achieve its aim of defending against virus infection. However, it can be an indirect result, a molecular marker or an indicator of symptom development. To address the relationship between PR-10 expression and symptom development, additional experiments will be required.

The RNA4-encoded putative ORF contains five potential in-frame initiation codons. Our results clearly demonstrated that the first AUG in the P31 ORF was used for expression of P31, but the second and third AUG may have weak functions in expressing an incomplete protein if the first AUG was shifted. This was indirectly demonstrated by the GUS activities. Such lower accumulation levels are probably due to a nucleotide sequence upstream of the AUG codon [53], which can down-regulate ribosome scanning and initiation at the first AUG. We viewed other proteins encoded by BNYVV, including RdRp, CP, P14 and P26, and found that these proteins also had more than one potential initiation codon in the RNA’s 5′-leader sequence [54]. This may be an expression strategy. As observed, if P31 was incomplete, then, regardless of the spontaneous deletion mutant, it could not induce *PR-10* up-regulation or severe systemic symptoms in *N. benthamiana*. Thus, the totality of P31 is necessary for viral pathogenicity and induction of *PR-10* expression [13].

Cys with its thiol side-chain often participates in enzymatic reactions. Because of its high reactivity, the thiol group of cysteine has numerous biological functions [55]. Disulfide bonds are common to many extracellular proteins, where they presumably serve to stabilize the native conformation, and the improved stability can be translated in practical terms as better resistance to environmental extremes [56,57]. The zinc finger is a structural motif of small proteins that is characterized by the coordination of one or more zinc ions to stabilize the fold [58]. In general, zinc fingers coordinate zinc ions with a combination of Cys and histidine residues, and recently different structures have been found [59]. *Beet soil borne mosaic virus* (BSBMV) is a member of *Benyvirus* and possesses a similar genomic organization to BNYVV [60]. A putative protein, P32, is encoded by BSBMV RNA-4 (GenBank: FJ424610.2) and has a similar function to P31. For instance, it is involved in symptom expression and viral transmission through its vector *P. betae*[61]. A sequence alignment of P31 and P32 showed that both were rich in Cys, about 6.03% and 6.36%, respectively, and, interestingly, 12 Cys shared the same locations. Based on structural predictions, several of them may link to form disulfide bonds, and if these links are broken, then P31 is probably easily degraded. In the N-terminal sequence of P31, there is a Cys7Cys9His19Cys24 motif. Although it is not predicted to result in a zinc finger, we hypothesize that it, or another region, has a similar functional domain. P31 is involved in silencing suppression in roots [13]. P14 and P16, which are found in BNYVV and *Tobacco rattle virus*, have the ability to suppress transgene silencing specifically in roots. All of these are cysteine-rich proteins, so we suspect that Cys may play an important role in root suppression.

In our results, plants infected by Cys mutants showed different relationships between *PR-10* expression and symptom appearance. The most prominent one, C161A, showed a seven-fold higher expression level of *PR-10* than that found with a BN3 infection, but it could not induce serious symptoms. We suspect that the region containing Cys 161 was important for symptom appearance but had less impact on the interaction with factors that up-regulated *PR-10* expression. Accompanying *PR-10* up-regulation, P31 can induce some symptoms, but the Cys mutants showed that P31 had more than one functional motif. Determining how these motifs function will require further experiments.

Proteins that perform biological functions should interact with other protein(s), and the amino acid Trp is more conserved within protein hotspot sites [62,63]. Different residues in proteins affect their structure and function [64]. The three-dimensional structure of P31 has not been reported, so we cannot determine the function of Trp in the protein structure. The Trp mutants resulted in the loss of *PR-10* induction and disease symptoms, suggesting that Trp may be involved in protein–protein interactions between virus and host.

## Materials and methods

### Preparation of RNA4 mutants

DNA manipulation and cloning were performed as described by Sambrook *et al*. [65]. The mutants used in the experiment were produced by PCR-based, oligonucleotide-directed mutagenesis based on full-length RNA4 cDNA clone pUOF1-6 [13]. Amino acid scanning mutants were also obtained by site-directed mutagenesis. All constructs were examined for sequence integrity.

### Plants, viruses and viral inoculations

*T. expansa* and *N. benthamiana* were used for viral propagation and the observation of symptom phenotypes [13,21]. The BNVYY laboratory isolated BN3 (RNA1 + 2 + 3) was used [66]. Unless otherwise stated, virus isolates were propagated in inoculated leaves of *T. expansa*. Infectious cDNA clones were linearized by *Xba*I and transcribed at 37Â°C for 2 h with a T7 RNA polymerase kit as described by the manufacturer (Promega). The leaves of test plants were inoculated with the *in vitro*-synthesized transcripts from each cDNA clone mixed with BN3. Local lesions generally appeared at 5–8 dpi, while systemic symptoms in *N. benthamiana* appeared at 10–12 dpi.

### RNA extraction and reverse transcription (RT) PCR

Total RNA was extracted from 0.2 g of top leaves of tested *N. benthamiana* and used for RT-PCR detection [67]. RT-PCR was conducted using Moloney Murine Leukemia Virus Reverse Transcriptase as described by the manufacturer (Promega). cDNA was synthesized from 3 Î¼g total RNA with oligo(dT) primer. The primers used for BNYVV detection were described previously [39]. Each experiment was repeated at least two times, and four plants were inoculated with each mutant. The total RNA extracted from each plant was tested separately by RT-PCR and qPCR.

### qPCR

cDNA synthesized by RT was used for qPCR. The qPCR was performed using a Bio-RAD CFX96 Touchâ„¢ Real-Time PCR Detection System according to the manufacturer’s instructions, and the primer pairs used for qPCR are found in Table 1. The level of PP2A transcript amplified with primer pair PP2A-F and PP2A-R was used as an internal control. Quantification and statistical analysis of the qPCR results were performed using the software Bio-Rad CFX Manager Version 3.1 (Bio-Rad Laboratories Inc.).

### GUS histochemistry

Freshly tested plant samples were incubated for 4–12 h at 37Â°C in the darkness in sufficient X-Gluc staining buffer (2 mM 5-bromo-4-chloro-3-indolyl Î²-D-glucuronic acid, 100 mM sodium phosphate buffer at pH 7.0, 10 mM EDTA, 0.5 mM potassium ferricyanide, 0.5 mM potassium ferrocyanide and 0.1% Triton X-100). Then, chlorophyll was removed with 70% (v/v) ethanol at room temperature.

### Cloning of a new pathogenesis-related protein 10 gene from *N. benthamiana*

During a database (http://solgenomics.net/) search with *Nicotiana tabacum PR-10* (GenBank ID:JQ041907), a sequence from *N. benthamiana* highly homologous to *NtPR-10* was identified. A pair of specific primers (PR10-forward: 5′-ATGGGTGTCACTACCTATACTCATG-3′ and PR10-reverse: 5′-TTAAACATAGAGAGAAGGATTAGCG-3′) were synthesized based on the newly identified sequence to clone the putative PR-10 gene (*NbPR-10*). Total RNA was isolated from young *N. benthamiana* leaves infected by BN3 + 4 and was reverse-transcribed to cDNA. The PCR product was gel purified and ligated into a pMD19-T vector (Takara) to obtain pMD–NbPR10 and confirmed through DNA sequencing.

### YTH assays

The yeast strains, *Saccharomyces cerevisiae* AH109 and Y187, and the yeast vectors, pGBKT7 and pGADT7, as well as the positive control plasmids, pGBKT7-T and pGADT7-P53, were used for YTH analyses (Clontech). The fragments of NbPR-10 and P31 were ligated to pGBKT7 and pGADT7, respectively. Yeast transformations were conducted using the small-scale lithium acetate method. YTH assays were performed using the Matchmaker GAL4 Two-Hybrid System3 (Clontech), according to the manufacturer’s protocols. Co-transformants were plated on synthetic defined (SD) minimal medium minus adenine, histidine, leucine and tryptophan (SD/–Ade/–His/–Leu/–Trp).

## Competing interests

The authors declare that they have no competing interests.

## Authors’ contributions

WQW, HYF and NJ performed the experiments. YW, ZYZ, YLZ, XBW, DWL, JLY contributed reagents/materials/analysis tools. CGH conceived of the study and participated in its design and coordination. WQW, YW and CGH wrote the manuscript. All authors read and approved the final manuscript.
